# Long-term adaptation of *Escherichia coli* to methanogenic co-culture enhanced succinate production from crude glycerol

**DOI:** 10.1007/s10295-017-1994-0

**Published:** 2017-12-12

**Authors:** Nam Yeun Kim, Su Nyung Kim, Ok Bin Kim

**Affiliations:** 1 Department of Life Science, Ewha Womans University, 52, Ewhayeodae-gil, Seodaemun-gu, Seoul, 03760 Republic of Korea; 2 Interdisciplinary Program of EcoCreative, The Graduate School, Ewha Womans University, 52, Ewhayeodae-gil, Seodaemun-gu, Seoul, 03760 Republic of Korea

**Keywords:** Long-term adaptation, *Escherichia coli*, *Methanobacterium formicicum*, Succinate, Crude glycerol

## Abstract

*Escherichia coli* can hardly grow anaerobically on glycerol without exogenous electron acceptor. The formate-consuming methanogen *Methanobacterium formicicum* plays a role as a living electron acceptor in glycerol fermentation of *E*. *coli*. Wild-type and mutant *E*. *coli* strains were screened for succinate production using glycerol in a co-culture with *M*. *formicicum*. Subsequently, *E*. *coli* was adapted to glycerol fermentation over 39 rounds (273 days) by successive co-culture with *M*. *formicicum*. The adapted *E*. *coli* (19.9 mM) produced twice as much succinate as non-adapted *E*. *coli* (9.7 mM) and 62% more methane. This study demonstrated improved succinate production from waste glycerol using an adapted wild-type strain of *E*. *coli* with wild-type *M*. *formicicum*, which is more useful than genetically modified strains. Crude glycerol, an economical feedstock, was used for the cultivation. Furthermore, the increase in methane production by *M*. *formicicum* during co-culture with adapted *E*. *coli* illustrated the possibility of energy-saving effects for the fermentation process.

**Electronic supplementary material:**

The online version of this article (10.1007/s10295-017-1994-0) contains supplementary material, which is available to authorized users.

## Introduction

Crude glycerol is an excellent feedstock candidate that is discarded as waste from biodiesel production [4, 5]. The waste glycerol from biodiesel production accounts for approximately 10% (w/w), or approximately 14 million tons [1, 19]. The bioconversion of glycerol to chemical building blocks is important to support the biofuel industry, as well as to lower production costs for succinate. Succinate is a multi-purpose platform chemical that can be produced from renewable biomass by microbes [8, 20, 25]. The global succinate market has experienced steady growth and reached 157.2 million USD and 58.5 kilotons in 2015 [9].

Various groups studied the succinate production from glycerol. The microbes known to produce succinate from glycerol are *Anaerobiospirillum succiniciproducens* [6], *Pasteurellaceae* family species and *Mannheimia succiniciproducens* [17], *Actinobacillus succinogenes* [24], *Yarrowia lipolytica* [29], *Corynebacterium glutamicum* [8], and *Escherichia coli*. Several studies have investigated the succinate production from glycerol using *E*. *coli* strains. Dharmadi et al. [4] focused on the pH-dependent mechanism of the *E*. *coli* fermentation of glycerol. They found that the production of CO_2_ from formate was required for increased glycerol consumption and succinate production. Blankschien et al. [2] improved succinate production by blocking the synthesis of competing by-products and the expression of *Lactococcuslactis* pyruvate carboxylase, which drives the generation of succinate from pyruvate production. Zhang et al. [30] engineered three gene mutations (pck*, *ptsI* ^−^, *pflB* ^−^) in *E*. *coli* ATCC 8739. The redirection of carbon flow in the engineered genes resulted in the maximum succinate yield. Soellner et al. [18] constructed a double mutant of *E*. *coli* (∆*pykA*, ∆*pykF*), from which a fast-growing strain was selected. In the selected strain, the third mutation in PEP carboxylase was found. Most recently, Li et al. [7] engineered an *E*. *coli* strain (*ldhA* ^−^, *pflB* ^−^, pck*) and performed two-stage fermentation that lead to an enhanced succinate production. In addition, *A*. *succinogenes* also enhanced succinate production from glycerol in the presence of dimethyl sulfoxide (DMSO) under controlled continuous microaerobic culture [16].

The mainstream approach of genetic engineering has generally adopted strategies for glucose fermentation, i.e., the elimination of competing pathways with the adjustment of the redox-balance and strengthening of the C_3_ to C_4_ branch, combined with process engineering to overcome the intrinsic redox imbalance [23]. *E*. *coli* strains rarely grow with glycerol in anaerobic conditions in the absence of an external electron acceptor: glycerol is imported by glycerol facilitator (GlpF), activated by glycerol kinase (GlpK) with ATP consumption, and oxidized to dihydroxyacetone phosphate (DHAP), whereby menaquinone (MQ) is reduced to menaquinol (MQH_2_) (Fig. S1). MQH_2_ emerges as every glycerol utilized, which must be recycled, therefore anaerobic growth on glycerol requires additional electron acceptors such as nitrate, DMSO, trimethylamine N-oxide (TMAO), or fumarate [22], and the amount of endogenous fumarate is not sufficient to recycle MQH_2_ to MQ. To overcome the redox imbalance of glycerol fermentation, Richter and Gescher [12] introduced the co-culture of *E*. *coli* and *Methanobacterium formicicum*, which uses formate in addition to H_2_–CO_2_ as its energy sources [15]. Glycerol fermentation and succinate production were higher in the co-cultures than in *E*. *coli* monocultures [12].

Our study screened co-cultures of several strains of *E*. *coli* (wild-type and genetically modified strains) with *M*. *formicicum*. We then adapted the *E*. *coli* to co-culture with *M*. *formicicum* in glycerol fermentation for 273 days. The long-term adapted *E*. *coli* developed in the present study demonstrated approximately twofold higher succinate levels than the non-adapted *E*. *coli* during crude glycerol fermentation.

## Materials and methods

### Strains and culture

*E*. *coli* K-12 strain MG1655 was used as the wild-type, and *E*. *coli* K-12 BW25113 gene knockout mutants were purchased from the National BioResource Project (National Institute of Genetics, Japan). *M*. *formicicum* JF-1 was obtained from the Leibniz Institute German Type Culture Collection (DSMZ, Germany). *E*. *coli* cells were anaerobically grown at 37 °C in Luria Broth (Affymetrix inc., USA), and kanamycin (30 µg/mL) was included for mutant *E*. *coli* strains. *M*. *formicicum* was anaerobically cultivated at 37 °C in DSMZ 119 medium. *E*. *coli* and *M*. *formicicum* were cultivated up to OD_600_ 1.20 and 0.27, respectively.

The adaptation medium contained 3 mM KH_2_PO_4_, 1 mM K_2_HPO_4_, 4 mM NH_4_Cl, 5 mM KCl, 6 mM NaCl, 1 mM MgCl_2_, 21 mM HCO_3_Na, 5 mM CO_3_Na_2_, 0.2 mM of sodium ascorbate, 5.1 mM CaCl_2_, 10 mL NB trace mineral solution [3], 1.0 mL selenite-tungstate solution (13 mM NaOH, 17 μM Na_2_SeO_3_, and 12 μM Na_2_WO_4_), 10 mL vitamin solution (DSMZ, media 141), 0.1% (w/v) yeast extract, 1 mM cysteine, and 2 µM resazurin. The pH value was adjusted to 7.0. To adapt *E*. *coli* on glycerol fermentation with *M*. *formicicum*, the co-cultivation of *E*. *coli* and *M*. *formicicum* was continuously sub-cultured until the 39th round, where each co-cultivation took 7 days. The co-culture was performed in 100 mL adaptation medium with 70 mM glycerol in 250-mL rubber-stoppered infusion bottles and cultivated anaerobically under a sterile 80% H_2_ + 20% CO_2_ gas mixture at 37 °C. Twenty percent of the co-culture pre-stage was inoculated into fresh medium. To this, additional 20% *M*. *formicicum* (v/v) that was cultured in DSMZ 119 medium was inoculated.

The crude glycerol fermentation medium contained 1.5 mM KH_2_PO_4_, 2.3 mM K_2_HPO_4_, 9.4 mM NH_4_Cl, 2 mM MgSO_4_, 2 mM CaCl_2_, 38.8 mM NaCl, 0.01 mM FeSO_4_, 20 mM HCO_3_Na, trace element solution SL-10 (DSMZ, media 320), 10 mL vitamin solution (DSMZ, media 141), 0.1% (w/v) yeast extract, 0.2% (w/v) casitone, 1.7 mM cysteine, 1.3 mM Na_2_S, and 2 µM resazurin. Ten percent *E*. *coli* (v/v) and 30% *M*. *formicicum* (v/v) were inoculated in the medium with 80 mM crude glycerol (AEKYUNG PETROCHEMICAL CO. LTD., Korea) (Table S1), and cultivated at 37 °C for 4 days anaerobically under a sterile 80% N_2_ + 20% CO_2_ gas mixture. DMSO (50 mM) was used to test the effect of an electron acceptor.

### HPLC analysis

Substrates and products in the supernatant of 1-mL cultures were analyzed using a HPLC Hitachi LaChrom Elite system (Hitachi High Technologies, Japan), consisting of an L-2130 pump, an L-2350 column oven, and an L-2200 auto-sampler. Ten-microliter samples were injected and separated using an Aminex HPX-87H ion-exclusion column (300 mm × 7.8 mm i.d., Bio-Rad, USA). The mobile phase was 4 mM H_2_SO_4_, which was pumped at a constant flow rate of 0.55 mL/min. The quantitative determination of substances was carried out using an L-2490 refractive index detector and an L-2400 UV detector (210 nm).

### Methane determination by GC

One-milliliter sample from the air space of culture was analyzed using a 6500GC System (YL Instruments, Korea). Gas samples were injected and separated using a Carboxen 1006 PLOT column (30 m × 0.53 mm i.d., Sigma-Aldrich Co. LLC., USA). The quantitative determination of methane was carried out using a flame ionization detector (YL Instruments, Korea).

### Cell growth analysis

Cell density of mixed *M*. *formicicum* and *E*. *coli* was determined at 600 nm wavelength using a UV/VIS spectrophotometer (X-ma1200, Human Corporation, Korea). The proliferation of *M*. *formicicum* and *E*. *coli* cells were quantitated by quantitative real time PCR (qRT-PCR), as described previously [28]. The *M*. *formicicum* primers, forward (5′- CGWAG GGAAG CTGTT AAGT-3′) and reverse (5′- TACCG TCGTC CACTC CTT-3′), and *E*. *coli* K-12 primers, forward (5′- ACTCC TACGG GAGGC AG-3′) and reverse (5′- GACTA CCAGG GTATC TAATC C-3′), were obtained from Cosmo Genetech (Korea); product sizes were 343 and 468 bp, respectively. Standard curves for the qRT-PCR were obtained by using plasmids that included partial 16S rRNA genes of *M*. *formicicum* M.o.H. and *E*. *coli* K-12, which were provided by the Environmental Bioprocess Engineering Laboratory (POSTECH, Korea). For standard curves of *M*. *formicicum* and *E*. *coli*, 16S rRNA gene copy numbers ranged from 2.6 × 10^9^ to 2.6 × 10^2^ and from 2.5 × 10^9^ to 2.5 × 10^2^, respectively. Logarithmic values of different 16S rRNA gene amounts were plotted against the threshold cycle (CT) number from each result. The linear range of the standard curve was selected based on the *R* ^2^ value of slopes, which were 0.9964 and 0.9945 for *M*. *formicicum* and *E*. *coli*, respectively. The average slope and average intercept were calculated, and the resulting equation was used to quantify 16S rRNA gene abundance in samples. CT values of each sample were compared to the corresponding standard curve. Genomic DNA was extracted using NucleoSpin Microbial DNA kits (Macherey–Nagel, Germany) and used as a template for qRT-PCR. Total reaction volume was 20 μL included 400 nM each primer in SensiFAST™ SYBR No-ROX Mix (Bioline, USA). The qRT-PCR analysis used 40 cycles of denaturation at 95 °C for 10 s, annealing at 60 °C for 20 s, and extension at 72 °C for 20 s and was performed in a Corbett Research Rotor-Gen RG-3000A (Qiagen, Germany) and the Rotor-Gene software, version 6.1.93.

### Statistics

Statistical analyses were performed using PASW Statistics 18. Unpaired two-tailed student’s *t* tests were performed to analyze the data. Statistical significance was defined as *P* < 0.05.

## Results and discussion

### Co-culture of wild-type or mutant *E*. *coli* with *M*. *formicicum*

In the co-culture of wild-type *E*. *coli* with *M*. *formicicum*, glycerol consumption and succinate production were highly improved by 12-fold and 8-fold, respectively, in comparison with the single cultivation (Table 1). Accordingly, other fermentation products were also increased, but formate was used up by *M*. *formicicum* (Table 1). To select the most suitable *E*. *coli* strain for succinate production during co-fermentation, mutant strains with specific gene deletions (*pflB*, *adhE*, *pta*, or *ackA*) involved in each competitive pathway against succinate production were cultivated with *M*. *formicicum* under the conditions of glycerol fermentation without exogenous electron acceptors (Table S2). The *pflB* (pyruvate formate lyase) mutant, for which all pathways other than that of succinate production were blocked, served as a negative control. The *adhE* (alcohol dehydrogenase) mutant did not grow at all in either single- or co-culture, indicating that ethanol production is an unavoidable step. In *pta* (phosphate acetyltransferase) and *ackA* (acetate kinase) mutants of *E*. *coli*, the acetate production was blocked, with the co-cultures of *M*. *formicicum* designed to produce succinate without both formate and acetate. In the co-culture, the *pta* or *ackA E*. *coli* mutants could grow to some extent, but the glycerol consumption was low, and the succinate production level did not exceed half that of wild-type *E*. *coli* co-culture (Table S2).

**Table 1:** Glycerol fermentation in single- and co-culture of *Escherichia coli* with *Methanobacterium formicicum*

	Consumed (mM)	Produced (mM)				Cell number (mL^−1^)	
	Glycerol	Succinate	Formate	Acetate	Ethanol	*E. coli*	*M. formicicum*
Single culture							
*E. coli*	4.6 ± 1.6	1.0 ± 0.1	10.7 ± 3.5	1.7 ± 0.9	9.6 ± 2.0	3.4 × 10^8^ ± 0.4 × 10^8^	ND
Co-culture							
*E. coli* *M. formicicum*	53 ± 12.8^a^	8.0 ± 0.7^a^	0	7.3 ± 1.0	47.2 ± 16.1	1.1 × 10^9^ ± 0.2 × 10^9^	2.1 × 10^8^ ± 1.2 × 10^8^

Product analysis and cell growth were determined after 7 days of fermentation. Values report means ± standard deviations for three replicates

^a^Value means a significant difference between single- and co-culture (unpaired samples *t* test, *P* < 0.05). ND, not determined

Collectively, co-culture of *E*. *coli* mutants with *M*. *formicicum* did not efficaciously improve succinate production. Among *E*. *coli* strains, we found that wild-type was the best strain for co-culturing with *M*. *formicicum*. For this reason, wild-type *E*. *coli* was performed into long-term adaptation for succinate production. Moreover, the use of wild-type (non-GMO) microbes is an incomparably large advantage for industrial applications.

### Crude glycerol fermentation by long-term adapted or non-adapted *E*. *coli*

Without an exogenous electron acceptor, *E*. *coli* hardly ferment glycerol. The co-cultivation of *E*. *coli* with *M*. *formicicum* allows *E*. *coli* to promote glycerol fermentation (Table 1), as reported by Richter and Gescher [12]. During co-cultivation for 7 days, *E*. *coli* fermented 53 mM of glycerol and produced 8 mM of succinate, while formate was completely consumed by *M*. *formicicum* (Table 1). This co-cultivation of *E*. *coli* with *M*. *formicicum* was adapted via 39 successive rounds. The adaptation lasted 273 days in total, in which each co-cultivation was carried out in batch culture for 1 week, and successively inoculated.

**Fig. 1:**
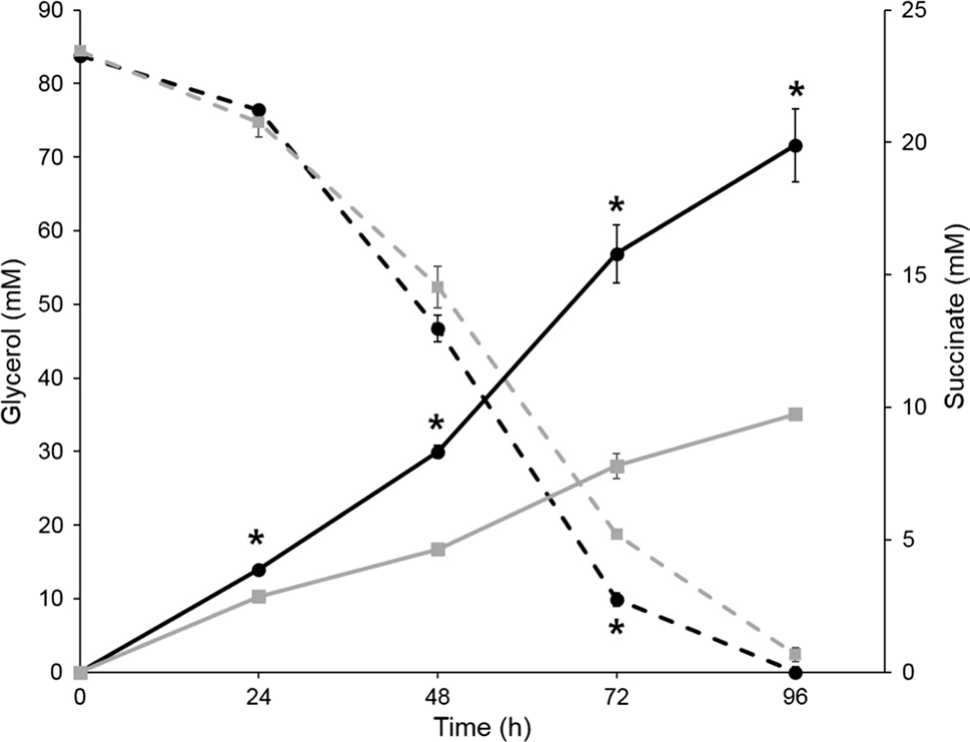
Succinate production from crude glycerol fermentation during co-culture of *Escherichia coli* with *Methanobacterium formicicum*. Solid lines represent succinate production, and dotted lines represent glycerol consumption by adapted (black circles) and non-adapted (gray squares) *E*. *coli*. *Value for the adapted *E*. *coli* was significantly different from that for the non-adapted *E. coli* (unpaired samples *t* test, *P* < 0.05). Plotted points report the means, and error bars report the standard deviations for independent samples taken in triplicate

The *E*. *coli* adapted to glycerol fermentation exhibited a two-fold increase in succinate production (19.9 mM) over non-adapted *E*. *coli* (9.7 mM succinate) during crude glycerol fermentation for 96 h (4 days) (Fig. 1; Table 2). Twenty-four percent of the PEP (from 83.8 mM glycerol) was metabolized and reduced to succinate in the adapted *E*. *coli* co-culture, whereas only 12% of the PEP (from 82 mM glycerol) was reduced to succinate in the non-adapted *E. coli* co-culture (Fig. 1; Table 2; Fig. S1). Methane production was higher in the adapted co-culture (206978 ppm) than in the non-adapted (127552 ppm) (Table 2). Methane production is often followed by improved growth or substrate consumption rates of the primary carbon source consumers and methane can easily be collected for use as energy in fermentation processes [12, 27]. The adapted *E*. *coli* (14.0 mM) also exhibited improved succinate production in the presence of DMSO compared with non-adapted *E*. *coli* (7.2 mM), but co-culture with *M*. *formicicum* produced even more succinate (19.9 mM) (Table 2). Co-culture with *M*. *formicicum* permitted a higher crude glycerol consumption by *E*. *coli* than that of culture with DMSO, which indicated that formate consumption by *M*. *formicicum*, a living electron acceptor, is more advantageous than for the supply of the electron acceptor DMSO (Table 2). All product analysis data collected over the course of fermentation, including the pH values, are shown in Tables S3 and S4.

**Table 2:** Fermentative characteristics by adapted *Escherichia coli* on crude glycerol

	39th *E*. *coli* *M. formicicum*	1st *E*. *coli* *M. formicicum*	39th *E*. *coli* DMSO	1st *E. coli* DMSO	39th *E*. *coli* *M. formicicum* DMSO	1st *E*. *coli* *M. formicicum* DMSO
Fermentation						
Consumption (mM)						
Glycerol	83.8 ± 0.8	82.0 ± 1.0	46.3 ± 1.1	54.1 ± 1.1	78.5 ± 1.2	81.0 ± 1.4
Production (mM)						
Succinate	19.9 ± 1.4^a^	9.7 ± 0.2	14.0 ± 0.2^a^	7.2 ± 0	19.8 ± 0.5^a^	12.6 ± 0.4
Acetate	6.1 ± 1.8	5.3 ± 0.8	2.7 ± 0^a^	1.5 ± 0.1	7.0 ± 0.4	4.6 ± 1.4
Ethanol	56.9 ± 3.5^a^	74.8 ± 3.0	30.8 ± 0.1^a^	52.2 ± 1.6	47.3 ± 2.0^a^	67.5 ± 5.7
Formate	0	0	7.6 ± 0.3^a^	14.4 ± 1.2	0	0
Methane (ppm)	206977.7 ± 72056.8	127552.0 ± 11659.79	105.7 ± 81.5	528.0 ± 454.5	122956.7 ± 84541.3	62341.3 ± 3672.3
Growth						
Cell density (OD_600_)	1.33 ± 0.03	1.31 ± 0.02	1.01 ± 0.01^a^	1.10 ± 0	1.32 ± 0.02	1.41 ± 0.03
*E. coli* cell number (mL^−1^)	8.9 × 10^8^ ± 7.2 × 10^7^	8.5 × 10^8^ ± 1.5 × 10^8^	6.3 × 10^8^ ± 7.0 × 10^7a^	7.8 × 10^8^ ± 5.5 × 10^7^	8.6 × 10^8^ ± 4.0 × 10^7^	9.5 × 10^8^ ± 5.5 × 10^7^
*M. formicicum* cell number (mL^−1^)	7.8 × 10^7^ ± 1.3 × 10^7^	1.1 × 10^8^ ± 1.7 × 10^7^	ND	ND	6.3 × 10^7^ ± 4.1 × 10^6^	5.6 × 10^7^ ± 1.2 × 10^7^

*E. coli* was adapted to *Methanobacterium formicicum* by 39 successive rounds of cultivation on glycerol. The adapted (39th-round) or non-adapted (1st-round) *E. coli* was cultivated for 96 h on crude glycerol with *M. formicicum* or DMSO

Values report means ± standard deviations for three replicates

*ND* not determined, *39th E. coli* adapted *E. coli*, *1st E. coli* non-adapted *E. coli*

^a^Value means a significant difference between co-culture groups of adapted and non-adapted *E*. *coli* (unpaired samples *t* test, *P* < 0.05)

Under anaerobic conditions, *E*. *coli* cannot grow with glycerol as its sole carbon and energy source due to the metabolic dilemma of redox-balancing and energy acquisition (Fig S1). During conversion of glycerol to PEP, MQH_2_ and NADH_2_ are generated. For redox balancing, PEP could be reduced to succinate whereby MQH_2_ and NADH_2_ would be re-oxidized, but no ATP is generated in this pathway. For energy acquisition, PEP should be also degraded over pyruvate to acetate, formate, ethanol, or lactate. NADH_2_ is re-oxidized in the ethanol or lactate production, but this pathway requires additional electron acceptors like fumarate, DMSO, TMAO, or nitrate, of which reduction is coupled with oxidation of MQH_2_ [22]. Therefore, glycerol fermentation by *E*. *coli* alone was very slow and showed low levels of products (Table 1). The interspecies transfer of formate from *E*. *coli* to *M*. *formicicum* and consumption of formate by *M*. *formicicum* improved the glycerol fermentation by *E*. *coli*. Formate is derived from pyruvate in nonoxidative cleavage by PFL (pyruvate formate lyase), and reducing equivalents of the reaction remain in the formate [13]. Therefore, formate metabolism is a critical step for adjusting redox balance in fermentation [14]. In the absence of an exogenous electron acceptor, the formate channel FocA exports formate. As the external pH decreases, formate is re-imported by FocA, undergoes disproportionation into CO_2_ and H_2_ by cytoplasmic orientated formate hydrogenlyase (FHL), and the excess redox equivalents are released as H_2_ [11]. FHL complex is composed of formate dehydrogenase H (FDH-H) [HCOO^−^ → CO_2_ + H^+^ + 2e^−^, *E*′_0_ =  − 432 mV] and hydrogenase 3 [2H^+^ + 2e^−^ → H_2_, *E*′_0_ = − 414 mV] [10, 21]. Moreover, as the affinity of FDH-H of FHL to formate is very low (*K*_m_ = 26 mM) [14], therefore the FHL reaction was not sufficient to solve the redox imbalance of glycerol fermentation. *M. formicicum* also possesses a FocA-similar formate channel FdhC [26]. Therefore, in co-culture, formate exported by *E*. *coli* is imported into *M*. *formicicum* and is quickly used, which re-adjusts the equilibrium in the direction of fermentation. *M*. *formicicum* uses both H_2_ and formate as electron donors [15].

In conclusion, this study successfully adapted an *E*. *coli* strain for succinate production from waste glycerol by 39 successive rounds (273 days) of co-culture of *E*. *coli* and *M*. *formicicum*. The adapted *E*. *coli* produced twice amount of succinate in co-culture in comparison with the non-adapted *E*. *coli*, and the methane production by *M*. *formicicum* increased by 62%, whereas the glycerol consumption and cell growth were not increased, and the ethanol production decreased by 24%. We, therefore, speculated that the formate transfer from *E*. *coli* to *M*. *formicicum* became more efficient during the adaptation process, whereby the reduction step to ethanol production was decreased and the C_4_-branch enzymes, including PEP carboxylase, malate dehydrogenase, fumarase, and fumarate reductase, were upregulated. The basis of phenotypic changes should be further investigated by genome and transcriptome analyses.

## Electronic supplementary material

Below is the link to the electronic supplementary material.

Supplementary material 1 (PDF 324 kb)
