# Supplementary material for: Long-term adaptation of Escherichia coli to methanogenic co-culture enhanced succinate production from crude glycerol
Source: J Ind Microbiol Biotechnol. 2017 Dec 12;45(1):71–6. doi: 10.1007/s10295-017-1994-0 (PMC5762792; doi:10.1007/s10295-017-1994-0)
Supplement: Supplementary file 1 — Supplementary material 1 (PDF 324 kb) [file 10295_2017_1994_MOESM1_ESM.pdf]

Long-term adaptation of *Escherichia coli* to methanogenic co-culture enhanced succinate production from crude glycerol

<Journal of Industrial Microbiology and Biotechnology>

Nam Yeun Kim[1], Su Nyung Kim[2], Ok Bin Kim*[1,2]

[1]Department of Life Science and [2]Interdisciplinary Program of EcoCreative, Ewha Womans University, 52, Ewhayeodae-gil, Seodaemun-gu, Seoul 03760, Republic of Korea

*Correspondence to: Ok Bin Kim

E-mail: kimokbin@ewha.ac.kr

Figure S1. Predicted metabolic pathway for the co-cultivation of *E. coli* and *M. formicicum*. Black, the metabolic pathway in *E. coli*; red, the metabolic pathway in *M. formicicum*. Abbreviations used: Glyc, glycerol; GlpF, glycerol facilitator; GlpK, glycerol kinase; Glyc-3p, glycerol 3-phosphate; GlpA, B, C, glycerol 3-phosphate dehydrogenase A, B, C; MQH_2, reduced menaquinone; DHAP, dihydroxyacetone phosphate; PEP, phosphoenolpyruvate; Ppc, phosphoenolpyruvate carboxylase; OAA, oxaloacetate; Mdh, malate dehydrogenase; Mal, malate; Fum, fumarase; FrdA, B, C, D, fumarate reductase A, B, C, D; Suc, succinate; Pyr, pyruvate; LdhA, lactate dehydrogenase; Lac, lactate; PflB, formate acetyltransferase; Ac-CoA, acetyl-CoA; AdhE, aldehyde-alcohol dehydrogenase; EtOH, ethanol; Pta, phosphate acetyltransferase; AckA, acetate kinase; For, formate; Ace, acetate; FocA, FdhC, formate transporter; $F_{420}H_2$, reduced coenzyme F_{420}; MF, methanofuran; H_4MP, tetrahydromethanopterin; CoM, Coenzyme M; CoB, Coenzyme B.

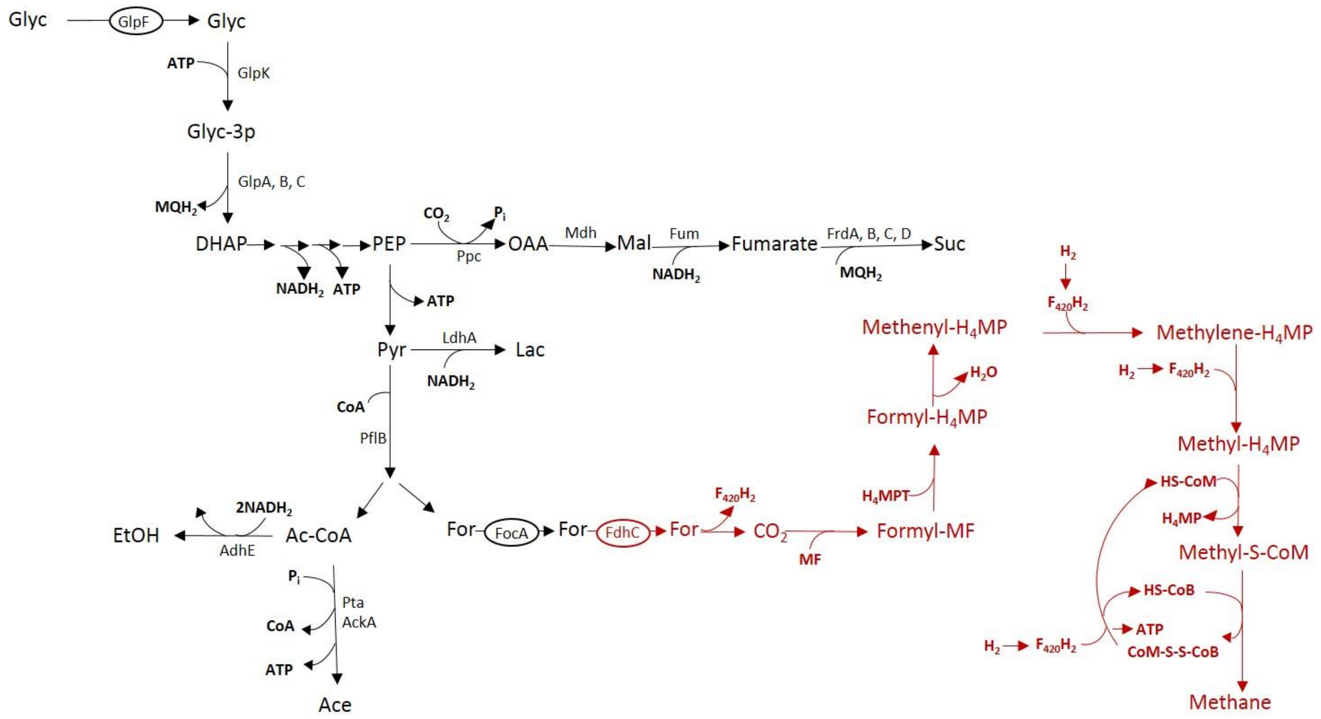

Table S1. Composition of crude glycerol

Purity, %	80min
Moisture,%	15 max
Ash, %	5 max
MONG, %	1.5 max
pH	4.5-6.0

Table S2. Glycerol fermentation in single- or co-culture of *E. coli* with *M. formicicum*. Product analysis and cell growth were determined after 7 d of fermentation.

		Consumption (mM)	Production (mM)				Cell number (T_0) (mL^{-1})	Cell number (T_7) (mL^{-1})	Cell number (T_0) (mL^{-1})	Cell number (T_7) (mL^{-1})
E. coli Strains	Culture condition	Glycerol	Succinate	Formate	Acetate	Ethanol	*E. coli*	*E. coli*	MF	MF
wild type	Single	4.6±1.6	1.0±0.1	10.7±3.5	1.7±0.9	9.6±2.0	5.0x10^{8}±1.6x10^{8}	3.4x10^{8}±0.4x10^{8}	ND	ND
	Co-culture	53±12.8	8.0±0.7	0	7.3±1.0	47.2±16.1	5.0x10^{8}±2.1x10^{8}	1.1x10^{9}±0.2x10^{9}	4.7x10^{7}±2.1x10^{7}	2.1x10^{8}±1.2x10^{8}
ΔpflB	Single	0.7±0.6	0.8±0.1	0	0.6±0.1	0	3.9x10^{8}±0.9x10^{8}	1.6x10^{8}±0.5x10^{8}	ND	ND
	Co-culture	1.9±2.3	1.8±0.2	0	0.2±0.4	6.4±0.6	4.5x10^{8}±1.6x10^{8}	2.4x10^{8}±0.2x10^{8}	3.8x10^{7}±0.9x10^{7}	6.7x10^{7}±2.1x10^{7}
ΔadhE	Single	0.8±0.2	0.4±0.1	2.6±0.6	1.8±0.5	0	3.9x10^{8}±0.6x10^{8}	6.8x10^{7}±3.6x10^{7}	ND	ND
	Co-culture	1.0±1.1	0.5±0.2	0	2.3±0.6	0	3.5x10^{8}±1.2x10^{8}	1.4x10^{8}±0.6x10^{8}	3.2x10^{7}±0	5.6x10^{7}±2.8x10^{7}
Δpta	Single	5.7±0.7	1.3±0.2	8.5±1.8	0	12.5±2.1	4.0x10^{8}±1.2x10^{8}	2.0x10^{8}±0.7x10^{8}	ND	ND
	Co-culture	18.7±5.1	3.4±0.4	0	2.4±1.2	23.0±6.7	1.8x10^{8}±0.9x10^{8}	4.8x10^{8}±3.2x10^{8}	2.5x10^{7}±1.1x10^{7}	1.3x10^{8}±0.7x10^{8}
ΔackA	Single	7.8±1.2	0.8±0	13.3±1.8	1.6±0.4	13.3±1.9	2.0x10^{8}±0.9x10^{8}	2.9x10^{8}±1.3x10^{8}	ND	ND
	Co-culture	28.5±2.3	1.9±0.1	0	5.2±0.9	35.8±3.6	2.3x10^{8}±0.8x10^{8}	7.3x10^{8}±1.1x10^{8}	2.8x10^{7}±1.7x10^{7}	1.6x10^{8}±0.2x10^{8}

Values report means ± standard deviations for 3 replicates.

Wild type MG1655; ΔpflB, *pflB* knockout MG1655; ΔadhE, *adhE* knockout MG1655; Δpta, *pta* knockout MG1655; ΔackA, *ackA* knockout MG1655

Table S3. Fermentative characteristics of adapted *E. coli* on crude glycerol. *E. coli* was adapted to *M. formicicum via* 39 successive rounds of cultivation on glycerol. The adapted (39th-round) or non-adapted (1st-round) *E. coli* was cultivated for 96 h on crude glycerol with *M. formicicum* or DMSO. Succinate production (A), crude glycerol consumption (B), and growth by the co-cultivation of *M. formicicum* with *E. coli* (C).

(A) Succinate production (mM)

Time (h)	39th *E. coli* *M. formicicum*	1st *E. coli* *M. formicicum*	39th *E. coli* DMSO	1st *E. coli* DMSO	39th *E. coli* *M. formicicum* DMSO	1st *E. coli* *M. formicicum* DMSO
0	0	0	0	0	0	0
24	3.9±0.1	2.9±0.1	4.4±0.1	2.7±0.2	4.9±0	3.5±0.3
48	8.3±0.2	4.7±0.1	8.7±0.2	5.1±0.1	10.2±0.1	6.6±0.4
72	15.8±1.1	7.8±0.5	12.2±0.1	6.4±0.1	16.2±0.2	10.7±0.4
96	19.9±1.4	9.7±0.2	14.0±0.2	7.2±0	19.8±0.5	12.6±0.4

(B) Crude-glycerin consumption (mM)

Time (h)	39th *E. coli* *M. formicicum*	1st *E. coli* *M. formicicum*	39th *E. coli* DMSO	1st *E. coli* DMSO	39th *E. coli* *M. formicicum* DMSO	1st *E. coli* *M. formicicum* DMSO
0	0	0	0	0	0	0
24	7.4±0.2	9.7±2.0	7.4±0.6	8.3±0.6	17.4±1.1	17.9±4.6

48	37.1±1.8	32.0±2.8	23.1±2.7	25.6±0.8	40.4±1.4	45.2±1.8
72	73.9±0.9	65.6±0.4	40.5±1.3	45.3±3.2	70.0±2.2	72.1±1.0
96	83.8±0.8	82.0±1.0	46.3±1.1	54.1±1.1	78.5±1.2	81.0±1.4

(C) Optical density (OD_{600}) and cell numbers (mL^{-1})

Time (h)	39th *E. coli* *M. formicicum*	1st *E. coli* *M. formicicum*	39th *E. coli* DMSO	1st *E. coli* DMSO	39th *E. coli* *M. formicicum* DMSO	1st *E. coli* *M. formicicum* DMSO
0	0.36±0.01	0.37±0.01	0.27±0.01	0.28±0.01	0.35±0.02	0.36±0.02
24	0.67±0.02	0.73±0.02	0.77±0.02	0.87±0.01	0.85±0.01	0.98±0.03
48	1.08±0.01	1.06±0.01	0.98±0.02	1.09±0.01	1.16±0.02	1.28±0.02
72	1.32±0.04	1.25±0.02	1.01±0.01	1.12±0.01	1.31±0.02	1.39±0.02
96	1.33±0.03	1.31±0.02	1.01±0.01	1.10±0	1.32±0.02	1.41±0.03

Time (h)		39th *E. coli* *M. formicicum*	1st *E. coli* *M. formicicum*	39th *E. coli* DMSO	1st *E. coli* DMSO	39th *E. coli* *M. formicicum* DMSO	1st *E. coli* *M. formicicum* DMSO
0	*E. coli*	$2.8 \times 10^8 \pm 7.2 \times 10^7$	$3.5 \times 10^8 \pm 1.5 \times 10^7$	$1.3 \times 10^8 \pm 6.8 \times 10^7$	$2.2 \times 10^8 \pm 1.2 \times 10^7$	$2.2 \times 10^8 \pm 4.2 \times 10^7$	$2.9 \times 10^8 \pm 5.5 \times 10^7$
	MF	$4.3 \times 10^7 \pm 1.3 \times 10^7$	$4.4 \times 10^7 \pm 1.0 \times 10^7$	0	0	$3.5 \times 10^7 \pm 8.1 \times 10^6$	$4.1 \times 10^7 \pm 8.1 \times 10^6$

96	*E. coli*	$8.9x10^8±7.2x10^7$	$8.5x10^8±1.5x10^8$	$6.3x10^8±7.0x10^7$	$7.8x10^8±5.5x10^7$	$8.6x10^8±4.0x10^7$	$9.5x10^8±5.5x10^7$
	MF	$7.8x10^7±1.3x10^7$	$1.1x10^8±1.7x10^7$	0	0	$6.3x10^7±4.1x10^6$	$5.6x10^7±1.2x10^7$

Reported values are means ± standard deviations for 3 replicates. ND, not determined; 39th *E. coli*, adapted *E. coli*; 1st *E. coli*, non-adapted *E. coli*.

Table S4. pH evaluation and amounts of methane, formate, acetate, and ethanol

(A) pH

Time (h)	39th *E. coli* *M. formicicum*	1st *E. coli* *M. formicicum*	39th *E. coli* DMSO	1st *E. coli* DMSO	39th *E. coli* *M. formicicum* DMSO	1st *E. coli* *M. formicicum* DMSO
0	7.13	7.14	7.14	7.15	7.20	7.17
24	7.16	7.18	7.07	6.95	7.09	7.03
48	6.77	7.05	6.59	6.52	6.67	6.77
72	5.79	6.62	5.91	5.84	5.83	6.35
96	6.19	6.92	6.10	6.14	5.95	6.84

(B) Formate (mM)

Time (h)	39th *E. coli* *M. formicicum*	1st *E. coli* *M. formicicum*	39th *E. coli* DMSO	1st *E. coli* DMSO	39th *E. coli* *M. formicicum*	1st *E. coli* *M. formicicum*

					DMSO	DMSO
0	0	0	0	0	0	0
24	0	1.9±0.7	3.7±0.2	5.6±0.4	0	2.8±0.7
48	0	0	6.8±0.4	10.5±1.0	0	4.2±1.1
72	3.4±0.2	0	8.0±0.5	14.1±1.7	1.2±1.1	3.0±2.3
96	0	0	7.6±0.3	14.4±1.2	0	0

(C) Acetate (mM)

Time (h)	39th *E. coli* *M. formicicum*	1st *E. coli* *M. formicicum*	39th *E. coli* DMSO	1st *E. coli* DMSO	39th *E. coli* *M. formicicum* DMSO	1st *E. coli* *M. formicicum* DMSO
0	0	0	0	0	0	0
24	2.1±0.3	1.7±0.2	2.2±0.2	1.9±0.2	2.8±0.1	2.4±0.1
48	3.6±0.6	2.2±0.3	2.2±0.1	1.5±0.1	4.4±0.2	3.1±0.1

72	5.4±1.3	3.5±0.5	2.4±0	1.3±0.1	5.9±0.2	3.6±0.9
96	6.1±1.8	5.3±0.8	2.7±0	1.5±0.1	7.0±0.4	4.6±1.4

(D) Ethanol (mM)

Time (h)	39th *E. coli* *M. formicicum*	1st *E. coli* *M. formicicum*	39th *E. coli* DMSO	1st *E. coli* DMSO	39th *E. coli* *M. formicicum* DMSO	1st *E. coli* *M. formicicum* DMSO
0	0	0	0	0	0	0
24	20.1±2.9	25.3±2.9	14.3±4.7	18.3±5.5	19.0±0.8	21.7±4.3
48	28.4±4.1	39.2±2.8	18.6±1.5	29.2±1.3	24.3±1.5	37.6±1.3
72	54.3±2.1	65.3±2.3	26.9±0.2	46.3±1.8	44.6±1.0	63.0±1.9
96	56.9±3.5	74.8±3.0	30.8±0.1	52.2±1.6	47.3±2.0	67.5±5.7

Reported values are means ± standard deviations for 3 replicates. ND, not determined; 39th *E. coli*, adapted *E. coli*; 1st *E. coli*, non-adapted *E. coli*.
